# Economic costs of gender inequality in health and the labor market: India's untapped potential

**DOI:** 10.3389/fpubh.2023.1067940

**Published:** 2023-01-30

**Authors:** Aqeel Khan, Shiraz Khan, Muhammad Azhar Khan, Khalid Zaman, Haroon ur Rashid Khan, Arieff Salleh Bin Rosman, Yasinta Indrianti, Abidin Ali Hassan

**Affiliations:** ^1^Faculty of Social Sciences and Humanities, Universiti Teknologi Malaysia (UTM), Skudai, Johor, Malaysia; ^2^Department of Management Sciences, The University of Haripur, Haripur, Pakistan; ^3^Department of Economics, The University of Haripur, Haripur, Pakistan; ^4^Faculty of Business, The University of Wollongong in Dubai, Dubai, United Arab Emirates; ^5^Center of Research for Fiqh Science and Technology (CFIRST), Universiti Teknologi Malaysia (UTM), Skudai, Johor, Malaysia; ^6^Entrepreneurship Department, Podomoro University, Jakarta, Indonesia; ^7^Department of Economics and Finance, College of Business Administration, University of Bahrain, Sakheer, Bahrain

**Keywords:** women's autonomy, gender equality, female under-5 mortality, female labor force participation rate, precarious employment, seemingly unrelated regression

## Introduction

The United Nations Sustainable Development Agenda–2030 gives member states a roadmap to achieve 17 developmental goals (1). The United Nations has generally stated that the SDGs can be achieved if women are given equal rights in all public and private domains and are empowered by equal work opportunities, equitable health care, and high-quality education. The United Nations SDG-5 was created expressly to achieve gender equality and highlighted eliminating all types of physical and domestic abuse, sexual exploitation, forced marriages, early marriages, trafficking, and female genital mutilation. Furthermore, the SDG suggested that women may be empowered through equal sharing of information and communication technology, allowing women to be more aware of their living rights and take better care of their health, education, and access to finance. Women should be allowed and encouraged to participate in economic and political decision-making, reproductive healthcare services, and economic resources that enable them to contribute to the country's economic progress (2).

India is one of the world's most populous and fastest-growing economies, although it is ranked moderately and weakly in many global indexes of advancement. According to the UNICEF (3), gender inequality is severe in India. The healthcare system reacts ineffectively to reducing newborn and under-five female mortality rates. As a result, females die at a higher rate than boys, and girls drop out of school at a higher rate than boys (4, 5). Furthermore, adolescent boys have more independence than girls, including the ability to get better health and education facilities and better occupations. Girls had significant challenges in obtaining healthcare, education, and better occupations, while forced and early marriage contributed to widespread poverty and uneven economic resources (6–8). Through various socioeconomic and healthcare policies, the government continues to move toward significant women-centered reforms to reduce gender disparity.

There is a wealth of literature on the role of women's empowerment in achieving socioeconomic and environmental sustainability, however, a limited number of studies investigate the root causes and repercussions of gender disparity, particularly in India, filling a vacuum in the literature by including a wide range of healthcare, labor market, and economic issues throughout the country. Ved et al. (9) discovered widespread gender disparities in education, health, and the labor market in India, highlighting the need to reform economic policies to promote gender equality. Choudhury and Kumar (10) discovered that the biggest impediments to gender equality in North East India are culture and customary practices that prohibit women from full participation in society. Mishra and Bhardwaj (11) discovered considerable economic disparity in India due to educational disparities between the rural and urban sectors. A more fair allocation of resources would aid in the reduction of economic disparity in a country. According to Atal and Foster (12), impoverished urban Indian women are severely stressed due to social exclusion from economic resources. Datta and Sahu (13) concluded that microfinance institutions actively created jobs and empowered women in rural India. Gupta (14) underlined the need to promote women's work through NGO assistance, allowing women to achieve immediate livelihood through training and education. Women should be equipped with information technology, government regulations, increased networking with their clientele, and innovative thinking encouraged by Non-Governmental Organizations (NGOs) in Uttarakhand, India. ViggKushwah et al. (15) argued that financial help from the government might achieve gender equality and empower women. The government should make easy access to financing and equitable economic opportunities available to minimize gender disparity in India.

The following research concerns remain unanswered and are attempted to be addressed in this study. First, to what degree do the country's colossal healthcare costs impede women's equality in the country's sustainability agenda? This question primarily focuses on a female under-five mortality rate, leading to female child healthcare difficulties such as death due to insufficient healthcare facilities and gender-bias laws. Second, whether increasing female labor force participation helps to minimize vulnerable employment and supports women's economic autonomy. This issue is about labor market rigidities in which female labor force participation has dropped significantly due to gender-bias regulations, increasing female vulnerability in the labor market. Third, whether increasing general prices and women's access to financing affects women's empowerment. This issue suggests that a persistent increase in the general price level and a lack of women's access to the financial market had a significant impact on household budgets and women's political autonomy, resulting in lower economic growth.

The study investigates the influence of a high female under-five mortality rate, vulnerable employment, and female labor force participation on women's autonomy and economic growth. Further, the study explores the role of finance in promoting women's political autonomy and economic growth. At last, the study evaluates changes in the general price level that raise household expenditures while decreasing the country's economic activity. These goals have been established to exercise women's roles in the country's economic growth by establishing gender equality in the healthcare sector and labor force to carry the country's sustainable development policies ahead. The study employed various statistical techniques, including SUR for analyzing parameter estimates, Granger causality estimates for a causal relationship between the candidate variables, and Variance Decomposition Analysis (VDA) for an inter-temporal forecast relationship between the stated factors.

The study's contribution is to employ two basic models to examine the causes and implications of gender inequality in the Indian economy. The first model would evaluate the significant drivers of women's empowerment in a country, including, women's business and law index score (designated as women's empowerment), female under-five mortality rate (designated for healthcare reforms), vulnerable female employment, financial development, and price level fluctuations. Earlier studies were mostly limited to either primary research or utilizing a few macroeconomic parameters to analyze gender reforms in a nation. However, there is still a push to consider more feminist elements alongside financial ones to fill the research vacuum (16, 17). The second model evaluates the repercussions of gender variation, which primarily influenced its economic activity. The study used the country's per capita income as a response variable, which depends upon women's empowerment, female labor force participation rate (designated for educational reforms), female vulnerable employment, under-5 female's mortality rate, financial development, and price level. Earlier research focused on achieving social and economic sustainability through other factors (18, 19). These studies enable us to propose a realistic approach to economic growth by encouraging women in future economic and public policies.

### Theoretical framework

The models' theoretical aspects are offered here for concept development. In feminist views, the study offered 'finance aspect' theory, 'social aspect' theory, and 'healthcare modernization' theory. Financial aspect theory, allowing women to enter the financial sector aids in achieving equal rights for women to advance in a gender-neutral economy (20). However, the proposed relocation is not without complications. The absence of political autonomy and restrictions to entry for women in the financial sector make it difficult for them to achieve sovereignty in the capital market (21). According to the social aspect theory, price increases cause a slew of social concerns that women, in particular, should suffer as responsible home agents to manage their family budget. The growing societal difficulties around food price increases have a detrimental influence on women's progress toward development (22). According to the healthcare modernization paradigm, women have equal and universal rights to healthcare services. Gender-biased labor and healthcare regulations, on the other hand, harmed women's dreams of living in a fairy tale since they faced fragile job status, poor working conditions, and high female newborn death rates. Furthermore, uneven healthcare facilities and the digitization of healthcare infrastructure promote diverse inequality, harming working-class women (23).

The research carries on to another model, Model-II, referred to as probable 'consequences' of gender disparity in a nation. Based on the development of Model-II, the study presented three possible theoretical frameworks. First, Solow (24) created a growth model based on technology, labor, and capital. Solow growth models did not distinguish between male and female labor forces and assumed equal participation, increasing economic growth. In reality, males earned larger financial benefits and higher incomes than women in the dominant male society and pro-men labor ideologies, with a differential influence on economic development (25). Second, it is evident that more expenditure on healthcare infrastructure eventually leads to increased economic growth through the channel of financial development. This influence, however, should not be sustained unless healthcare services are extended to society's most disadvantaged sector. Under-5 female mortality rates are one example of the severe increases caused by a lack of equitable healthcare opportunities in rural areas compared to metropolitan areas (26), and finally, societal growth model refers to a condition in which economic prospects have been harmed due to uneven work opportunities and rising prices. The weak female labor force causes many societal ills, including increased poverty and economic disparity, and it forces ordinary people to live in misery (27). Furthermore, the rise in food costs has resulted in a slew of social difficulties that have hampered the country's economic progress (28).

## Materials and methods

### Data and measures

The data is taken from the World Development Indicators published by the World Bank (29). The study employed an index of women in business and law, which served as a proxy for women's autonomy that is used as a response variable in the study. Other variables include the under-5 female mortality rate, the female labor force participation rate, and vulnerable female employment, served as explanatory variables of the study. Furthermore, the study included three controlled variables: domestic credit to the private sector, served as a proxy for financial development, GDP per capita, and inflation.

### Econometric models

The research established its first model, which aids in determining the various 'causes' of gender disparity in a nation, based on the three mentioned feminist theories. The model building process is a step-by-step procedure for comprehending its pragmatic modeling, i.e.,

- First step: Women's emancipation is preceded by financial growth
  (1) $$\begin{array}{l} WABL=\beta_{0}+\beta_{1}FD+\beta_{2}X+\varepsilon \end{array}$$
  $\frac{\partial WABL}{\partial FD}>0$, if and only if domestic financing accessible to the private sector regards women as equal economic agents to men,
  $\frac{\partial WABL}{\partial FD}<0$, If and only if domestic financing accessible to the private sector is skewed toward men.
  Where WABL shows women's autonomy in business and law, FD shows financial development, and X shows other factors.
- Second Step: Price changes are subject to changes in women's autonomy
  (2) $$\begin{array}{l} WABL=\beta_{0}+\beta_{1}INF+\beta_{2}X+\varepsilon \end{array}$$
  $\frac{\partial WABL}{\partial INF}>0$, if and only if the percentage of changes in food costs is less than the predicted growth in female labor-force wages, whereas
  $\frac{\partial WABL}{\partial INF}<0$, If and only if the percentage of changes in food costs is greater than the predicted growth in female labor-force wages.
  Where INF shows inflation.
- Third Step: Women's autonomy was impacted by their employment and healthcare status
  (3) $$\begin{array}{l} WABL=\beta_{0}+\beta_{1}FLFPR+\beta_{2}FVE+\beta_{3}FU5MR+\beta_{4}X\\ +\varepsilon \end{array}$$
  $\frac{\partial WABL}{\partial FLFPR}>0$, $\frac{\partial WABL}{\partial FLFPR}<0$, the increase in women's autonomy is related to their positive contribution to the labor force, whereas the reduction in women's autonomy is due to their negative contribution.
  $\frac{\partial WABL}{\partial FVE}<0$, because of the significant susceptibility of female employment in the labor force, the male-biased labor force advancement causes a loss in women's autonomy.
  $\frac{\partial WABL}{\partial FU5MR}<0$, the high prevalence of female infant death under five would reduce women's autonomy.

It is assumed that a lack of easy access to finance for women, a significant shift in food costs, and unequal labor and healthcare economic policies all contribute to a reduction in women's empowerment in a country. At the same time, the opposite is true for equitable women's justice and proportional women's growth, both of which contribute to economic prosperity.

The research built its second model based on the above theories to estimate the potential 'consequences' of gender disparity in a nation. The following is a step-by-step procedure:

- First step: Women's self-determination in the development model
  (4) $$\begin{array}{l} GDPPC=\gamma_{0}+\gamma_{1}WABL+\beta_{2}X+\varepsilon \end{array}$$
  $\frac{\partial GDPPC}{\partial WABL}>0$, The circumstance alluded to the positive benefits of including women in political issues to progress toward equal growth.
  Where GDPPC shows GDP per capita.
- Second Step: Infrastructure and financial development in the healthcare sector as part of a growth model
  (5) $$\begin{array}{l} GDPPC=\gamma_{0}+\gamma_{1}FU5MR+\gamma_{2}FD+\gamma_{3}X+\varepsilon \end{array}$$
  $\frac{\partial GDPPC}{\partial FU5MR}<0$, female under-5 death rates are higher, which has a detrimental influence on the country's development objective, whereas,
  $\frac{\partial GDPPC}{\partial FD}>0$, the stronger the financial growth, the more opportunities for equity-based economic ventures open up.
- Third Step: Social Dimension of economic development
  (6) $$\begin{array}{l} GDPPC=\gamma_{0}+\gamma_{1}FLFPR+\gamma_{2}FVE+\beta_{3}INF+\beta_{4}X+\varepsilon \end{array}$$
  $\frac{\partial GDPPC}{\partial FLFPR}>0$, the greater the proportion of females in the labor force, the better the country's income on an equitable basis.
  $\frac{\partial GDPPC}{\partial FVE}<0$, when women's precarious work threatens their livelihood and causes them to live in deplorable conditions, it has a detrimental influence on the country's per capita GDP, and
  $\frac{\partial GDPPC}{\partial INF}>0$, and $\frac{\partial GDPPC}{\partial INF}>0$, single-digit inflation raises the quantity of supply, which raises the country's revenue. In contrast, the social problems faced by the poor become worse when inflation rises, increasing poverty and inequality. As a result, the country's per capita income falls.

Using data from 1975 to 2019, the first model was constructed to determine the factors influencing women's empowerment in the Indian economy. The second model depicts the link between women's empowerment and economic growth, i.e.,

### Model-I: Factors influencing women's empowerment


(7) $$\begin{array}{l} WABL=\beta_{0}+\beta_{1}FLFPR+\beta_{2}FVE+\beta_{3}FU5MR+\beta_{4}FD\\ +\beta_{5}INF+\varepsilon \end{array}$$

Where FLFPR shows female labor force participation rate, FVE shows vulnerable female employment, and FU5MR shows female under-5 mortality rate.

### Model -II: Women's empowerment and economic growth


(8) $$\begin{array}{l} GDPPC=\gamma_{0}+\gamma_{1}WABL+\gamma_{2}FLFPR+\gamma_{3}FVE+\gamma_{4}FU5MR\\ +\gamma_{5}FD+\gamma_{6}INF+\varepsilon \end{array}$$

Where FLFPR shows female labor force participation rate, FVE shows vulnerable female employment, and FU5MR shows female under-5 mortality rate.

The contribution of women to economic growth is expected to be favorable. This influence, however, may be maintained and long-lasting provided the country's healthcare infrastructure and female labor force reforms allow for equal growth. Finally, the social infrastructure should be strong enough to counteract vulnerability and food inflation to achieve widespread growth. Table 1 shows the theoretical expectation of the variables under the two given models.

**Table 1:** List of variables and theoretical expectations.

**Variables**	**Symbol**	**Measurement**	**Expected Sign**	**Theoretical Support**
**Model -I: Factors influencing women's empowerment**				
Women business and law index score (represented by women's autonomy)	WABL	Index Scale, 1 indicating low autonomy and 100 indicating maximum autonomy.	–	–
**Independent variables**				
Female under-5 mortality rate	FU5MR	Per 1,000 live births	Negative	Healthcare modernization theory
Female vulnerable employment	FVE	FVE as % of total female employment	Negative	Labor market stringent theory
Female labor force participation rate	FLFPR	% of female population, 15+ years old (ILO estimates)	Positive	
Inflation, consumer price index	INF	Annual %	Negative	Social aspect theory
Domestic credit to the private sector (represented by financial development)	FD	% of GDP	Positive	Financial inclusion/support theory
**Model -II: Women's empowerment and economic growth**				
GDP per capita	GDPPC	Constant 2010 US$	–	–
**Independent variables**				
Women business and law index score (represented by women's autonomy)	WABL	Index Scale, 1 indicating low autonomy and 100 indicating maximum autonomy.	Positive	Feminist Solow growth theory
Female under-5 mortality rate	FU5MR	per 1,000 live births	Negative	Health-led financial growth theory
Female vulnerable employment	FVE	% of female employment	Negative	Labor market stringent theory
Female labor force participation rate	FLFPR	% of female population, 15+ years old	Positive	
Inflation, consumer price index	INF	Annual %	Positive	Societal growth theory
Domestic credit to the private sector (represented by financial development)	FD	% of GDP	Positive	Finance-led growth theory

World Bank (29).

The study employed the SUR technique to acquire robust parameter estimates, which helps to suggest appropriate policy implications in the particular context, based on the wide theoretical backing for the models. The SUR approach was introduced by Zellner (30), and it is based on the generalization of linear regression equations with various response variables and unique explanatory factors. Because of the unique structure of the variance-covariance matrix, the SUR model may consist of several SUR equations (SURE), which are regarded as the most efficient estimator than Ordinary Least Squares (OLS). SUR is a predictive model used to evaluate several regression equations concurrently. It may be used to handle the difficulty of autocorrelation, a form of association that happens when a predictive method's residuals (forecast defects) are associated across space or various units. Autocorrelation may be a concern since it can lead to erroneous estimations of the model's values and can also impact the accuracy of the predictions. One method in which SUR solves the difficulty of autocorrelation is by permitting the error terms for each regressor to be associated with one another. This enables the system to consider that the error terms may be connected, which may improve the precision of the estimations. Furthermore, SUR permits the incorporation of identity effects, which may assist in adjusting for advanced features that may be connected with the latent variables and that might alter the results. SUR is especially beneficial when the variables being examined are linked across various equations or when the standard errors for the analytical calculations are interrelated. It may also be used when analyzing the factors have multiple measurement units.

## Results

The study used the SUR modeling approach to determine functional relationships between the candidate variables in the two separate models represented by equations (1) and (2). The SUR estimates for the equation (1) are shown in Table 2. The findings reveal that women's autonomy in business and law is harmed by the female under-5 mortality rate and vulnerable female employment, with coefficient estimates of −0.116 *p* < 0.000 and −1.095 *p* < 0.000, respectively. With an estimated elasticity value of −0.304 percentage points, the elasticity estimations confirmed the less elastic relationship between females under-5 mortality rate. The association between vulnerable female employment and women's autonomy, on the other hand, is more elastic, with an estimated elasticity value of −1.627 percentage points. The elasticity estimates show that vulnerable female employment impacts women's autonomy more than female under-5 mortality rates.

**Table 2:** SUR estimates for equations (1) and (2).

**Variables**	**Coefficient**	**Elasticity Estimates^a^ (%)**	**Std. Err**.	**z-statistics**	**P>z**
FU5MR	−0.116^***^	−0.304	0.013	−8.870	0.000
FVE	−1.095^***^	−1.627	0.262	−4.170	0.000
FLFPR	0.561^*^	0.269	0.314	1.790	0.074
INF	−0.088	−0.014	0.094	−0.930	0.350
FD	−0.177^***^	−0.096	0.054	−3.240	0.001
Constant	159.241^***^	–	14.312	11.130	0.000
**Diagnostic test estimates**					
RMSE	1.510	R^2^	0.947	Chi-square	812.290
**SUR estimates for equation (2)**					
WABL	16.746^***^	1.089	3.806	4.400	0.000
FU5MR	−1.382^**^	−0.165	0.555	−2.490	0.013
FVE	−56.525^***^	−5.463	7.897	−7.160	0.000
FLFPR	1.336	0.042	8.302	0.160	0.872
INF	−0.716	−0.006	2.430	−0.290	0.768
FD	8.368^***^	0.298	1.551	5.390	0.000
Constant	4,754.579^***^	–	707.852	6.720	0.000
RMSE	38.583	R^2^	0.994	Chi-square	7,668.790

Dependent variable: WABL for equation (1) and GDPPC for equation (2).

*, ** and *** indicate that the corresponding coefficient is significant at 10, 5, and 1% levels.

Table 2 further showed that the rate of female labor force participation and financial development had varied effects on women's autonomy. With a coefficient estimate of 0.561, *p* < 0.074, a positive association was discovered between women's autonomy and female labor force participation. In contrast, a negative relationship was discovered between women's autonomy and financial development with a coefficient estimate of −0.177, *p* < 0.001. The elasticity estimations suggest that the stated factors have a less elastic relationship. Women's autonomy grows by 0.269 percentage points and falls by −0.096 percentage points with a 1% growth in the female labor force and financial development. The findings suggest that female labor force involvement is beneficial to achieving women's empowerment in a country. On the other hand, women's autonomy is harmed by a lack of easy access to finance. As a result, there is a growing need to incorporate women in decision-making to capitalize on financial resources and access the capital market to support their lifestyles (31, 32).

The SUR estimates for Equation 2 depicts the potential effects of gender disparity on the country's economic growth. According to the findings, the female under-5 mortality rate and vulnerable female employment negatively influence the country's GDP per capita. It implies that gender inequality in each of the aforementioned factors is one of the primary causes of women's revocation, which harms the country's economic progress. According to the elasticity estimates, a 1% rise in female under-5 mortality rates and vulnerable female employment reduces the country's economic development by −0.165 and −5.463%, respectively. Estimates reveal that a high female vulnerability in the labor force reduces the country's economic growth more than female under-5 death rates. Other findings indicate that women's autonomy in business and law institutions and easy access to financing have a beneficial influence on the country's per capita income. The elasticity estimations supported the one-to-one association between women's autonomy and per capita income in the country. In contrast, the link between financial development and a country's economic growth is less elastic. As a result, women's political autonomy positively impacts the female labor force market, allowing them to access the financial market and participate in economic activities.

Research questions focus on healthcare expenses, labor market rigidity, and access to funding for women's equality and empowerment in a particular nation. Investigating the first research question, data on female under-five mortality rates and healthcare facilities are analyzed to determine the extent to which healthcare costs impede women's equality in the country's sustainability agenda. This analysis concludes that high healthcare costs impede women's equality in the country's sustainability agenda, as evidenced by a high female under-five mortality rate and lack of access to healthcare facilities. To address the second research question, data on female labor force participation and vulnerable employment is analyzed to determine whether increasing female labor force participation helps to minimize vulnerable employment and supports women's economic autonomy. The conclusion of this analysis supports that there is a correlation between increasing female labor force participation and minimizing vulnerable employment and that this supports women's economic autonomy. To address the third research question, data on general prices, women's access to financing, and women's empowerment are analyzed to determine whether increasing general prices and limiting women's access to financing affects women's empowerment. This analysis concludes that increasing general prices and limiting women's access to financing significantly negatively impact women's empowerment, resulting in lower economic growth.

## Discussion

### Main findings

According to Annan et al. (33), women typically have a robust decision-making capacity to care for their household expenses and their children's health, which leads to better reproductive health care decisions and economic prosperity. Morsy (34) discovered that women are more likely to be excluded from the financial sector due to widespread gender disparities in educational attainment, asymmetric credit information, a growing share of state-owned banks, and a decreasing share of foreign-owned banks, all of which make women more vulnerable to access the financial market. Brixiová et al. (35) stated that easy access to capital would help women become entrepreneurs, empowering women to operate businesses efficiently. Jaim (36) discovered that women entrepreneurs have difficulty obtaining bank loans due to gendered policies imposed by banks to release the money to their spouses. This skewed policy is one of the hurdles to admission for women in the workplace.

Guilmoto et al. (5) concluded that excess female mortality due to inadequate economic prospects worsened gender inequities and a high fertility rate, resulting in a fall in women's status in India. According to Matilla-Santander et al. (37), precarious employment and growing unemployment significantly impact healthcare, and a legislative framework must stabilize it. According to Salman and Nowacka (38), the most current and digital services assist women in having access to finance, empowering women in both the public and private spheres.

### Policy implications

In India, women account for about half of the population. Signs of social development in India include the rise in women enrolling in scientific programmes at the undergraduate and graduate levels. In India, over 35% of all PhD recipients in the sciences are women, and 15% of the country's entire Research and Development (R&D) workforce is comprised of women (39). To encourage more women to pursue careers in Science, Technology, Engineering, and Mathematics (STEM) fields, the Indian government has launched several initiatives, i.e., “Beti Bachao, Beti Padhao” (Save the Daughter, Educate the Daughter): Indian Prime Minister Narendra Modi launched the stated programme on 22 January 2015, one of several government-led efforts to advance women's rights and prosperity in the country. It is an organization-wide initiative to stop declining child sex ratio data (40). “Mahila-E-Haat”: The Indian government established this program in 2016. It is a website that promotes women-owned businesses and those owned by self-help groups and non-governmental organizations (41). “Mahila Shakti Kendra”: In 2017, the government established the Mahila Shakti Kendra to provide rural women with access to education, jobs, media skills, health services, and nutrition programmes (42).

The Indian economy owes a great deal to the efforts of women business owners. Biocon Ltd. Chairman and Managing Director Dr Kiran Mazumdar-Shaw, an entrepreneur, (II) Ekta Kapoor, the head of creative development at Balaji Telefilms. Sunita Narain, an activist and environmentalist. The Managing Director of Microsoft India, Neelam Dhawan, and Naina Lai Kidwai among Fortune's list of the 50 Most Powerful Corporate Women in the World. The increasing number of successful female company owners in India shows that women can achieve the same level of success as men in the corporate world (43, 44).

The role of women in economic development can be more visible if and only if women become involved in economic and financial affairs. According to financial aspect theory, easy access to finance would allow women to get involved in financial problems and receive easy credit and loans for beginning their own business. The digitalization of economic policy, equal educational opportunities, labor market flexibility, and common pay regulations are a few major steps toward empowering women to be successful businesswomen. The social aspect theory is concerned with societal social norms, particularly those that pose impediments to women's advancement. It is time for people to adjust their ideas and regard women as economic actors since they work for their families' bread and butter and provide support to their families the same way that men do. Investing in women to engage them in the mainstream economic agenda will unleash their potential, allowing them to achieve self-sufficiency and sustainable development and escape the misery of their families. The healthcare modernization theory advocates equitable and straightforward access to healthcare facilities to all people, particularly women and children, who are largely excluded from the healthcare sustainability agenda due to insufficient healthcare spending. Opportunities for gender-equitable healthcare, violence prevention, elimination, and leadership development are likely to help the UN SDGs' goal and vision, changing for a gender-equitable growth model. Women and children's health has improved due to the equitable distribution of healthcare expenditure, allowing women to participate equally in the country's economic development as men.

In the modern era, public health and policy have not yet made significant investments in ongoing research to address gender disparities in health and the underrepresentation of women in policy decision-making. The only way to ensure that women have a place in India's labor force is to raise the proportion of women working in each sector. The government may provide financial incentives to businesses that recruit more women and provide them with on-the-job training to help them become skilled employees. To achieve gender equality, we need gender-sensitive public health policies that preserve girls' reproductive rights to healthcare throughout their lives. Investing in efforts to reduce gender-based atrocities is both moral and practical. The costs of health and social issues and financial burdens are high, highlighting the necessity for solid laws to safeguard women in the workplace. Through local awareness-raising initiatives that contribute to the reduction of gender inequality in a nation, make it a priority to give young women and girls an essential voice in community discussions alongside religious authorities, community activists, and a diverse range of other stakeholders. Make accessible to women programmes that seek to enhance their economic and political position by educating them about civic involvement and providing access to microfinance opportunities.

### Study limitations

The female labor force participation rate is calculated as the proportion of women aged 16 and up in the civilian non-institutional population working or actively looking for work. National labor force surveys or census data are familiar sources for this metric, and future studies may obtain it through the suggested national data sets. The research might be expanded to include other aspects directly bearing on women's empowerment, such as the scientific and technical elements, environmentally conscious actions, leadership characteristics, and practical training skills. In addition, the caveat in the study's generalizability becomes more apparent when looking at the South Asian region.

## Conclusions

The study aims to identify the possible causes and consequences of gender inequality in the Indian economy by using the time series data set from 1975 to 2019. The study developed two different SUR equations based on the different theoretical supports. The growth model discussed women's autonomy and developed health led financial growth theory and societal growth theory to reach conclusive findings. The results show that female under-5 mortality rate and precarious female employment are the negative outcome variables that decrease women's autonomy in business operation and law-making policies, resulting in a negative impact on the country's economic growth. Moreover, female labor force participation rate and financial development support women's autonomy and the country's per capita income in a country. Asymmetric information concerning credit and loan disbursement, agricultural transformation, and extension services led to rural women experiencing pay declines and healthcare-related concerns in India. Women empowered by technological advances, on the other hand, contribute to eradicating stereotypes in many development initiatives that swiftly worked to support their lifestyles efficiently. Microfinance and small-scale business may be readily created in the rural sector. Women can take out loans and start their businesses, altering their lives and making them healthier and more confident. Stringent regulatory changes and a strong governance framework should support women as economic and productive agents who participate equally in all public and private programs. Enforceable legislation and safety from violence allow people to participate in economic affairs by the human rights convention. Finally, gender equality is the human right to equal opportunity in education, health, and political representation, allowing women to channel their energies into projects that promote long-term development.

## Data availability statement

Publicly available datasets were analyzed in this study. This data can be found here: The data is freely available at World Development Indicators published by World Bank (29) at https://databank.worldbank.org/source/world-development-indicators.

## Author contributions

All authors listed have made a substantial, direct, and intellectual contribution to the work and approved it for publication.
